# Manufacturing of nanoliposomal extract from *Sargassum boveanum* algae and investigating its release behavior and antioxidant activity

**DOI:** 10.1002/fsn3.1306

**Published:** 2019-12-06

**Authors:** Davood Savaghebi, Mohsen Barzegar, Mohammad Reza Mozafari

**Affiliations:** ^1^ Department of Food Science and Technology Tarbiat Modares University Tehran Iran; ^2^ Australasian Nanoscience and Nanotechnology Initiative 8054 Monash University LPO Clayton Vic. Australia

**Keywords:** algal extract, antioxidant, encapsulation, nanoliposome, polyphenols

## Abstract

In this paper, the fabrication of algal extract‐loaded nanoliposomes was optimized based on the central composite response surface design. Different concentrations of phenolic compounds (500, 1,000, and 1,500 ppm) of algal extract and lecithin (0.5, 1.25, and 2% w/w) were applied for preparation of nanoliposomes at process temperatures of 30, 50, and 70°C. Dependent variables were zeta potential, entrapment efficiency, size, and particle size distribution. The particle size of the loaded nanoliposomes ranged from 86.6 to 118.7 nm and zeta potential from −37.3 to −50.7 mV. The optimal conditions were as follows: 0.5% lecithin, 30°C process temperature, and 1,313 ppm of the phenolic compounds extracted from algae. Under these conditions, the experimental entrapment efficiency of the phenolic compounds was 45.5 ± 1.2%. FTIR analysis has verified the encapsulation of algal extract in nanoliposomes. Algal extract phenolic compounds also increased phase transition temperature (*Tc*) of nanoliposomes (1.6°C to 6.3°C). Moreover, the thermo‐oxidative protection of nanoliposomes for the algal extract has been proved by examining the DSC thermograms. It has been demonstrated that the formulated nanoliposomes have a good stability during storage conditions, and they are able to control the release of phenolic compounds at different pH values. During the encapsulation process, the antioxidant activity of the algal extract has been maintained to an acceptable level. Consequently, algal extract‐loaded nanoliposomes can be used as a natural antioxidant in lipid‐based foods.

## Graphical abstract

In this paper, the fabrication of algal extract‐loaded nanoliposomes was optimized. The thermo‐oxidative protection of nanoliposomes for the algal extract has been proved by examining the DSC thermograms. It has been demonstrated that the formulated nanoliposomes have a good stability during storage conditions and have been able to control the release of phenolic compounds at different pH values. Algal extract‐loaded nanoliposomes can be used as natural antioxidants in lipid‐based foods.

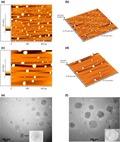

## 1 INTRODUCTION

Free radicals and reactive oxygen species are formed during oxidative damage of lipid‐containing foods. These molecules, in addition to undesirable effects on human health, lead to loss of nutritional value and acceptability of food products (Blomhoff, 2005). As a result, antioxidants are used to reduce and delay these undesirable effects. In recent years, consumption of synthetic antioxidants has been restricted due to carcinogenic and mutagenic effects. Therefore, the utilization of natural antioxidants has attracted much attention. Plant extracts have been recognized as natural antimicrobial and antioxidant agents, and their functional properties are attributed to the presence of secondary metabolites such as phenolic compounds. Phenolic compounds have many biological effects, including antioxidant, anti‐inflammatory, antimicrobial, antiviral, antiallergenic, and antithrombotic activities (Cox, Abu‐Ghannam, & Gupta, 2010; Faridi Esfanjani, Assadpour, & Jafari, 2018). However, direct use of the phenolic compounds as food preservatives is faced with some limitations. Adding these compounds to foods will result in adverse organoleptic effects in the product (Tavakoli, Hosseini, Jafari, & Katouzian, 2018). Moreover, food processing and storage conditions (exposure to high temperature, oxygen, and light) as well as low pH and presence of enzymes in the gastrointestinal tract can decompose the phenolic compounds. On the other hand, due to the low solubility and limited bioavailability of phenolic compounds and their interactions with other food ingredients, very high concentrations of plant extracts are necessary to prevent oxidation. Nowadays, a common way for incorporating phenolic‐rich extract in food products is encapsulation. Encapsulation methods can be applied for protection of phenolic compounds from decomposition and interactions with other food components, as well as for improving their bioavailability and accurate release in food systems (Bora, Ma, Li, & Liu, 2018).

Liposomes are among the most important lipid‐based carriers and are capable of encapsulating a wide range of materials with different polarity. These carriers are nontoxic, have a good biodegradability, and do not stimulate the immune system. Other advantages of liposomes include high encapsulation efficiency, controlled and targeted release, simple fabrication, and high stability. Nanoliposomes provide more surface area than liposomes and consequently provide more solubility and stability, higher bioavailability, and more accurate delivery to target areas. Due to amphipathic structure of nanoliposomes, antioxidant agents encapsulated in these lipid carriers can prevent the oxidation process initiation at water/oil interface in foodstuffs (Khorasani, Danaei, & Mozafari, 2018).

In recent years, some phenolic‐rich plant extracts have been successfully encapsulated with liposomes and were introduced as natural preservative in food systems (Pagnussatt et al., 2016; Pinilla, Noreña, & Brandelli, 2017; Rafiee, Barzegar, Sahari, & Maherani, 2017; Tavakoli et al., 2018).

Marine algae are exceptional sources of natural and bioactive compounds. Brown macro algae contains high amount of phenolic compounds such as catechins, phlorotannins, flavonoids, flavonols, and flavonol glycosides, and their organic extracts can be used as natural preservatives due to their potent antioxidant and antimicrobial activities (Cox et al., 2010; Zhao et al., 2018). Sargassum is the most diverse genus among Iranian macroalgae, and antioxidant properties of its extract have been proven (Kokabi & Yousefzadi, 2015; Lim et al., 2019). *Sargassum boveanum* is found in coastal waters of the Persian Gulf and can be considered as a natural and economical source of antioxidant compounds (Zahra, Mehranian, Vahabzadeh, & Sartavi, 2007).

Accordingly, this study aimed at determination of optimum conditions for encapsulating algal extract into nanoliposomes using response surface experiments and investigation of their main physicochemical properties. The feasibility of algal extract encapsulation in nanoliposomes was evaluated by Fourier‐transform infrared spectroscopy (FTIR). In addition, nanoliposomes stability, release behavior, and antioxidant activity of free and entrapped extract were studied.

## 2 MATERIALS AND METHODS

### 2.1 Plant material and chemicals

The brown seaweed *Sargassum boveanum* was collected in May 2017 on the coast of Bushehr, Iran (31 880 N, 49 217 E). The soybean lecithin (99%) was obtained from Acros Organics Chemical Co. All other chemicals and reagents were of analytical grade and supplied by Sigma‐Aldrich or Merck chemical Co.

### 2.2 Preparation of algal extract

The *S. boveanum* sample was washed thoroughly with distilled water to remove sea salts. After drying at 40°C in a vacuum oven, the algae were milled in an electric grinder and sieved through 0.5 mm. Ten grams of dry powder was mixed with 100 ml methanol for one hour in the orbital shaker (IKA, model KS4000i, Germany). The methanol extract was filtered through Whatmann No.1 filter paper and dried using a rotary evaporator. Subsequently, the crude extract was dissolved in distilled water and then fractionated sequentially by three solvents, dichloromethane (DCM), ethyl acetate, and n‐butanol according to the method of Lim, Cheong, Ooi, and Ang (2002). The solvents were removed from the extracts in a rotary evaporator. The resulting crude methanolic extract and its three fractions were determined for their total phenolic contents and antioxidant capacity as explained below.

### 2.3 Total phenolic content (TPC)

The Folin–Ciocalteu method was used to determine the total phenolic content of the extracts (Taga, Miller, & Pratt, 1984). 100 µl aliquot of algal extract was added to a test tube and mixed with 2.0 ml of 2% Na_2_CO_3_. After 2 min standing at room temperature, 100 µl of Folin–Ciocalteu reagent (1:1 diluted with distilled water) was added, and the mixture was shaken vigorously and placed in the dark for 30 min. The absorbance of each sample was measured at 720 nm with a spectrophotometer (Agilent Cary 60). The total phenolic contents are expressed as mg gallic acid equivalent per gram dry weight (GAE/gdw) of extract.

### 2.4 Liposome preparation using Mozafari method

Empty and loaded nanoliposomes were prepared using Mozafari method (Colas et al., 2007). First, the required amount of liposomal ingredients including algal extract phenolic compounds and lecithin, as indicated in Table 1, were weighted in a 50 ml glass beaker and then hydrated by adding 20 ml deionized water and 3% v/v glycerol. The mixture was stirred at 1,000 rpm on a hotplate shaker for 30 min at different process temperatures according to RSM design matrix (Table 1). Then to prepare nanoliposomes, the liposome suspension was subjected to sonication (1 min, 1s on and 1s off) using the probe sonicator (Sonicator 4,000, 20 kHz, maximum nominal power 600 W, high gain cylindrical titanium sonotrode of 19.1 mm in diameter; Misonix, Inc) at 80% of full power under controlled temperature (30 ± 5°C). Finally, in order to stabilize and anneal nanoliposomes, samples were kept at ambient temperature for 1 hr. In order to remove metallic particles mixed with samples during sonication the samples were centrifuged at 5,000 g for 20 min and then stored under nitrogen.

**Table 1:** Central composite design matrix

Runs	Independent variables			Responses			
	Lecithin Conc. (%w/w)	Temperature (^°^C)	Phenolic compounds Conc. (ppm)	PDI	Particle Size (nm)	Zeta potential (mV)	Entrapment efficiency (%)
1	1.25	50	1,000	0.257	96.26	−45.6	29.8
2	0.50	30	1,500	0.223	107.2	−38.4	50.2
3	0.50	30	1,500	0.245	104.9	−37.3	48.8
4	2.00	30	500	0.256	103.2	−48	13.4
5	2.00	30	1,500	0.242	101.7	−40.1	27.6
6	2.00	30	1,500	0.247	102.6	−39.4	29.7
7	1.25	50	1,500	0.241	101.9	−37.4	38.4
8	2.00	70	1,500	0.246	103.8	−38.8	31.7
9	0.50	30	500	0.25	101.7	−46.9	28.8
10	1.25	30	1,000	0.239	98.8	−45	31.7
11	1.25	50	1,000	0.242	98.22	−46.9	30.4
12	0.50	70	500	0.249	87.02	−50.7	28.5
13	1.25	30	1,000	0.245	97.98	−44.9	27.8
14	2.00	70	500	0.282	86.63	−50.6	13.4
15	0.50	50	1,000	0.252	100.4	−44.2	43.9
16	1.25	50	1,000	0.242	102.8	−45	32.6
17	1.25	50	1,000	0.247	101.6	−46.1	30.9
18	2.00	70	500	0.268	90.35	−50.2	15.4
19	1.25	50	1,000	0.246	99.62	−45.8	32.1
20	0.50	30	500	0.258	102.7	−47.1	27.2
21	0.50	70	500	0.251	90.1	−49.3	29.3
22	2.00	30	500	0.256	101.8	−49.3	12.3
23	0.50	50	1,000	0.246	104.6	−44.7	41.8
24	1.25	50	1,000	0.244	101.6	−45.4	31.8
25	2.00	50	1,000	0.274	98.8	−46.2	23.5
26	0.50	70	1,500	0.21	115.4	−38.4	46.4
27	1.25	70	1,000	0.254	97.8	−46.8	28.4
28	0.50	70	1,500	0.204	118.7	−37.4	48.3
29	1.25	70	1,000	0.245	93.1	−47.2	30.6
30	1.25	50	500	0.247	93.53	−48.5	19.1
31	1.25	50	1,500	0.243	103.5	−38.1	41.7
32	2.00	50	1,000	0.264	101.3	−47.1	21.7
33	1.25	50	500	0.251	90.83	−50	22.4
34	2.00	70	1,500	0.234	105.56	−39.4	33.5

### 2.5 Particle size and zeta potential

The particle size, PDI, and zeta potential of the nanoliposomal dispersions were determined by dynamic light scattering (DLS) using a Zetasizer Nano ZS instrument (Malvern Instruments) as described by Bouarab et al. (2014). All measurements were carried out at room temperature, assuming a medium viscosity of 1.0200 and medium refractive index of 1.335.

### 2.6 Determination of entrapment efficiency

The entrapment efficiency (EE) of nanoliposomes was determined based on the phenolic content using the Folin–Ciocalteu method (Madrigal‐Carballo et al., 2010). Nanoliposomes were sedimented by centrifugation at 65,000*g* for 1 hr (3‐30k; Sigma). Then, the amount of total phenolic content in the supernatant, containing free (unencapsulated) phenolic compounds, was measured and the entrapment efficiency was calculated from Equation 1.

(1) $$\text{EE}\%=\left(\left(P_{i}-P_{s}\right)/P_{i}\right)\times 100$$

where *P*_i_ is total amount of phenolic compounds and *P_s_* represents free phenolic compounds in the supernatant.

### 2.7 Transmission electron microscopy (TEM)

A quantity of 20 µl of the nanoliposomes suspension was placed on a carbon‐coated grid for 2 min. Then, the sample was negatively stained with 20 µl of 2% uranyl acetate for 1–2 min. After air‐drying at room temperature, the samples morphology was evaluated by TEM (Zeiss EM10C) operating at 100 kV (Ruozi et al., 2011).

### 2.8 Atomic force microscopy (AFM)

The noncontact AFM was employed to examine the morphology of the nanoliposomes samples. A drop of diluted nanoliposomes suspension was placed on freshly cleaved mica surface. The samples were dried for 1 hr at room temperature and then were visualized by an AFM (Autoprobe CP Research, Veeco) using a silicon probe (reflective side: Au; Chip size: 3.6 × 1.6 × 0.4 mm; force constant: 5.5–22.5 N/m; and resonant frequency: 190–325 kHz). The apparatus was equipped with one rectangular cantilever with the radius of 10 nm. The length, width, and thickness of cantilever were 100 ± 5 μm, 35 ± 5 μm, and 1.7–2.3 μm, respectively (Takahashi, Higashi, Ueda, Yamamoto, & Moribe, 2018).

### 2.9 FTIR

FTIR spectra of empty nanoliposomes, free extract, and loaded nanoliposomes were obtained using a Nicolet IR100 FTIR spectrometer (Thermo) from 4,000 and 400/cm wavenumbers. Plates were prepared by mixing samples with KBr before the analysis.

### 2.10 DSC analysis

The phase transition temperature (Tc) of nanoliposomes and oxidative stability of free and encapsulated algal extract were analyzed by DSC (Mettler Toledo DSC1, Switzerland). The Tc was determined by calorimetric scans from −30 to 50°C with a scan rate of 0.5°C/min. In order to evaluate oxidative stability, samples were placed under oxygen flow of 50 ml/min and were heated from 30 to 300°C as previously mentioned by Gortzi, Lalas, Chinou, and Tsaknis (2006).

### 2.11 Stability study of nanoliposomes suspensions

In order to determine the physical stability of empty and loaded nanoliposomes, changes in particle size, zeta potential, PDI, and entrapment efficiency were evaluated during storage at 4°C for 2 months under nitrogen atmosphere (Rafiee et al., 2017).

### 2.12 In vitro release

In vitro release of phenolic compounds from nanoliposomes was performed in acetate buffer (pH = 3 and 5) and phosphate‐buffered saline (pH = 7) according to Madrigal‐Carballo et al. (2010). Nanoliposomes containing phenolic compounds were isolated by centrifugation from the nanoliposomes suspensions and submerged in each of the above buffers. Then sealed tubes containing samples were kept for 14 days at room temperature and under continuous stirring (200 rpm). In the predetermined time intervals, the amounts of phenolic compounds were determined using the Folin–Ciocalteu method and the cumulative release (CR) percent of phenolic compounds from nanoliposomes was calculated using Equation 2.

(2) $$\text{CR(\textbackslash{}\%)}\;=\sum_{t=0}^{t}\frac{P_{t}}{P_{0}}\times 100$$

where *P_t_* is the amount of released phenolic compounds at time *t* and *P*_0_ is total amount of phenolic compounds in the formulation.

### 2.13 Determination of antioxidant activity

The antioxidant capacity of free and encapsulated extracts was evaluated by three antioxidant assays: DPPH radical scavenging activity (Sebaaly, Jraij, Fessi, Charcosset, & Greige‐Gerges, 2015), ABTS radical cation scavenging activity (Re et al., 1999), and ferric reducing antioxidant power (FRAP) (Benzie & Strain, 1996). Prior to analysis, the nanoliposomes were suspended in distilled water and placed in a shaker and agitated at 200 rpm for 2 hr to release the encapsulated phenolic compounds. Ascorbic acid and BHT were employed as positive controls in all tests, and the results were expressed as EC_50_ values.

### 2.14 Experimental design

A central composite response surface design with three factors and three levels was used to determine the optimum conditions for encapsulating of phenolic compounds in nanoliposomes. The independent variables were lecithin concentration (0.5, 1.25, and 2% w/w), temperature (30, 50, and 70°C), and the concentration of phenolic compounds (500, 1,000, and 1,500 ppm). polydispersity index (PDI), particle size (nm), zeta potential (mV), and entrapment efficiency were considered as responses of the design experiments. The experimental design was performed using Design‐Expert 10 and is listed in Table 1. The statistical differences between the means were evaluated by LSD test at *p* < .01 using SAS software.

## 3 RESULTS AND DISCUSSION

### 3.1 Total phenolic content and antioxidant capacity

Brown macroalgae and their organic extracts contain great amount of bioactive substances including polysaccharides, phenolic compounds, polyunsaturated fatty acids, proteins, pigments, and sterols. Among them phenolic compounds have a great contribution to the antioxidant activity of the algal extract (Zhao et al., 2018). In the present study, liquid–liquid extraction was performed to isolate high quantity of the phenolic compounds. The extraction yield, total phenolic contents, and antioxidant activity of the obtained extracts were measured (Table 2). Antioxidant activities of algal extracts were evaluated by DPPH^•^, ABTS^•^ **^+^** scavenging ability, and FRAP assays. Results were expressed by EC_50_ which represents the concentration of extract that is required to scavenge 50% of DPPH and ABTS free radicals, as well as 50% ferric reduction in the FRAP test. The less EC_50_ represents the higher antioxidant activity of the corresponding compound (Mishra, Ojha, & Chaudhury, 2012). As shown in Table 2, a positive correlation can be observed between total phenolic contents of algal extracts and their antioxidant activities. Ethyl acetate fraction had the highest TPC (542.6 ± 8.1mg GAE/ gdw extract) and antioxidant activity in comparison with other extracts. Consequently, it was selected for loading (encapsulation) in nanoliposomes.

**Table 2:** Extraction yield, total phenolic content, and antioxidant activity of algal extracts

Extracts	Extraction yield (%dw)	TPC (mg GAE/ gdw extract)	EC_50_ (ppm)		
			DPPH	ABTS	FRAP
Crude extract	8.2 ± 0.9^a*^	172.7 ± 3.4^d^	1,091.7 ± 30.1^d^	1,204.6 ± 54.9^d^	535.8 ± 26.9^d^
Fractions					
DCM	1.3 ± 0.2^c^	480.3 ± 8.7^b^	245.9 ± 10.9^b^	247.6 ± 11.1^b^	157.9 ± 9.1^b^
Ethyl acetate	0.4 ± 0.1^d^	542.6 ± 8.1^a^	171.4 ± 17.8^a^	219.5 ± 5.0^a^	129.2 ± 8.8^a^
Butanol	0.7 ± 0.1^d^	222.8 ± 7.6^c^	779.9 ± 13.2^c^	487.9 ± 15.7^c^	243.9 ± 9.7^c^
Water	5.7 ± 0.9^b^	73.4 ± 3.3^e^	1987.1 ± 62.4^e^	1556.4 ± 42.8^e^	1,381.7 ± 55.1^e^

Abbreviations: ABTS, 2,2'‐azino‐bis (3‐ethylbenzothiazoline‐6‐sulfonic acid); DPPH, 2,2‐diphenyl‐1‐picrylhydrazyl; and FRAP, ferric reducing antioxidant power.

* Different letters within each column represent significant differences among means determined by LSD test (*p* < .01); TPC: total phenolic content; GAE: gallic acid equivalent; dw: dry weight; EC_50_: the concentration of extract that is required to exert 50% antioxidant activity.

### 3.2 Characterization of nanoliposomes

The effect of different parameters on the properties of nanoliposomes was investigated, and the results are shown in Table 1. Particle size and PDI are among the important parameters in nanovesicles' stability and homogeneity (Sarabandi, Jafari, et al., 2019a). The particle size of loaded nanoliposomes was in the range of 86.6 to 118.7 nm. Furthermore, the formed nanoliposomes had a narrow particle size distribution and high uniformity, and their PDI values were less than 0.3. The other important factor in the physical stability of nanoliposomes in suspensions is zeta potential (ZP). Knowledge of the ZP of nanoliposomes sample can help to predict the fate of the formulation in vitro and in vivo. The magnitude of the ZP can be utilized to predict the stability and shelf life of the nanoliposomes. If the sample has a large negative or large positive ZP, then the particles will tend to repel each other and resist the formation of aggregates, hence implying a high level of stability. However, if the sample possesses a low ZP value, then there will be nothing to prevent the particles approaching each other and aggregate or fuse and eventually sediment (Larsson, Hill, & Duffy, 2012). As shown in Table 1, the zeta potential of loaded nanoliposomes varied between −37.3 and −50.7 mV. This large negative surface charge indicates that the nanoparticles are stable against aggregation and precipitation (Larsson et al., 2012). Generally, the presence of the phosphatidic acid in lecithin led to the formation of nanoliposomes with negative surface charge (Machado, Pinheiro, Vicente, Souza‐Soares, & Cerqueira, 2019). However, changes in the ZP values in this study are probably due to different concentrations of phenolic compounds among treatments and consequently the difference in the amount of phenolic compound absorption onto surface of liposomal membrane (Tavakoli et al., 2018). On the other hand, by phenolic compounds encapsulation, the particle size of nanoliposomes changes, which can affect the ZP values (Rasti, Jinap, Mozafari, & Yazid, 2012).

Entrapment efficiency of nanoliposomes depends on the type of wall material, ratio of core to wall material, encapsulation method, particles size, and total solid content (Tavakoli et al., 2018). In the present study, the entrapment efficiency improved by increasing the phenolic compounds concentrations but higher lecithin concentration decreased this parameter. By increasing lecithin content, a dense medium is created that restricts the free motion of phenolic compounds and reduces the entrapment efficiency (Rafiee et al., 2017). As shown in Table 1, the highest entrapment efficiency of phenolic compounds inside nanoliposomes was 50.2%. Pagnussatt et al., (2016) reported a similar entrapment efficiency for encapsulation of *Spirulina* sp. LEB‐18 phenolic extract (55%) into liposomes. Also, an entrapment efficiency of 47.5% was obtained for encapsulation of garlic extract in liposomes (Pinilla et al., 2017) which is in agreement with our result.

Independent variables involved in the fabrication of nanoliposomes were numerically optimized by response surface methodology (RSM). Considering the conditions necessary to obtain maximum entrapment efficiency of the phenolic compounds, the lowest PDI and the highest zeta potential of nanoliposomes, the independent variables were optimized as follows: 0.5% lecithin, 30°C, and 1,313 ppm of the phenolic compounds.

The nanoliposomes were prepared under the optimum conditions, and their properties were analyzed. Loaded nanoliposomes (LNL) showed an entrapment efficiency of 45.5%. After encapsulating the algal extracts in nanoliposomes, the particle size has increased from 79.1 nm to 104.3 nm. Moreover, loaded nanoliposomes had more homogeneity and uniformity in particle size and their PDI value (0.249) was slightly less than the empty vesicles (0.269). The zeta potential of loaded nanoliposomes was less than that of the empty vesicles (−41.2 mV vs. −49.8 mV). These results indicate that the empty nanoliposomes (ENL) are more stable than those containing the extract. The interaction between phospholipids and phenolic compounds determine the zeta potential of loaded nanoliposomes. Phenolic compounds not only can be placed inside the liposomes, but also can be absorbed to the surface of the liposome membrane. Therefore, phenolic compounds of algal extract can react with negative charged groups in liposome membrane and change the zeta potential (Machado et al., 2019). Besides, the placement of relatively positive compounds of algal extract around the surface of nanoliposomes can also increase the zeta potential towards positive values (Sarabandi, Sadeghi Mahoonak, Hamishehkar, Ghorbani, & Jafari, 2019b). Pinilla et al. (2017) observed a similar change in zeta potential (from −24.3 to −16.2) after encapsulation of garlic extract in nanoliposomes. Our results also were in agreement with Machado et al. (2019) who reported that liposomes containing the phenolic extracts of *Spirulina* LEB‐18 have zeta potential values lower than the liposomes without bioactive compounds (−11 mV vs. −46.7 mV).

### 3.3 Morphological analysis

**Figure 1:**
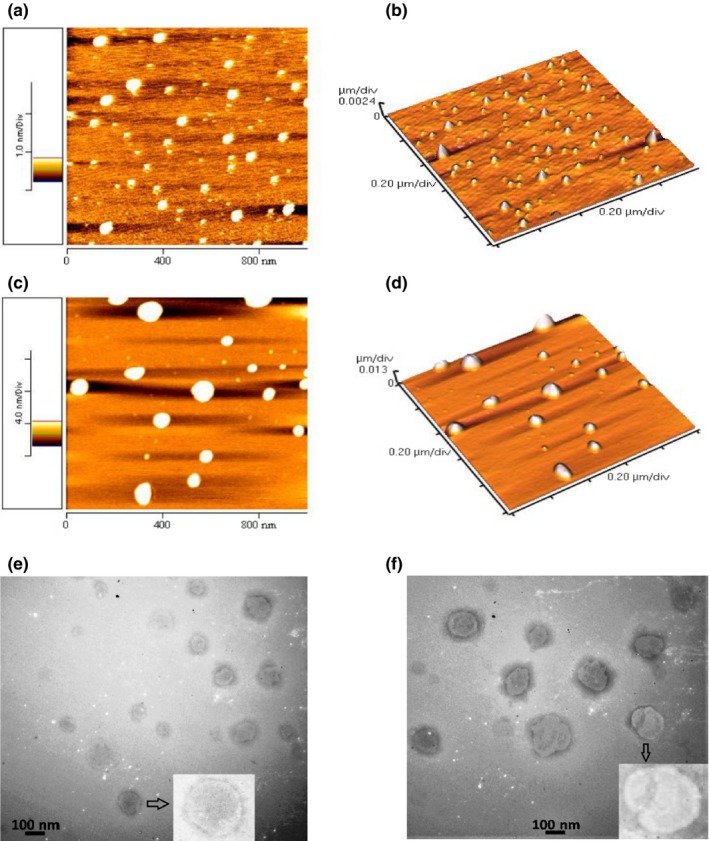
Two‐dimensional (a) and three‐dimensional (b) AFM micrographs of ENL (empty nanoliposomes containing 0.5% lecithin and manufactured at 30°C). Two‐dimensional (c) and three‐dimensional (d) AFM micrographs of LNL (loaded nanoliposomes containing 0.5% lecithin encapsulating 1,313 ppm phenolic compounds and manufactured at 30°C). TEM images of ENL (empty nanoliposomes formulated by 0.5% lecithin at 30°C) (e) and LNL (loaded nanoliposomes formulated by 0.5% lecithin and 1,313 ppm phenolic compounds at 30°C) (f)

The 3D morphology of the nanoliposomes was evaluated by atomic force microscopy (AFM). The AFM micrographs showed that the vesicles had uniform distribution and spherical shape (Figure 1). Moreover, the AFM images illustrated that the particle size of nanoliposomes increased after encapsulation, which was consistent with the results obtained through DLS analysis.

The microstructure of nanoliposomes was also investigated by transmission electron microscope (TEM). The TEM images of empty and loaded nanoliposomes under the optimized conditions are shown in Figure 1. As indicated in the figure, empty nanoliposomes had a particle size less than 100 nm and the particle size of loaded nanoliposomes was larger than 100 nm. These findings are in agreement with the DLS data. These vesicles had a bilayer structure and round shape, verifying that the prepared vesicles are nanoliposomes and not random aggregates of phospholipids.

### 3.4 FTIR analysis

**Figure 2:**
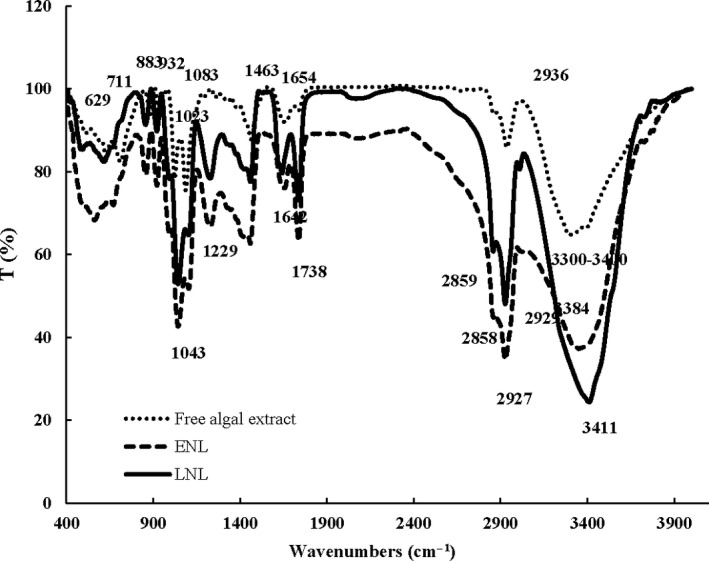
FT‐IR spectra of ENL (empty nanoliposomes formulated by 0.5% lecithin at 30°C), free algal extract, and LNL (loaded nanoliposomes formulated by 0.5% lecithin and 1,313 ppm phenolic compounds at 30°C)

FTIR spectroscopy was employed to verify algal extract encapsulation in nanoliposomes. The FTIR spectra of empty nanoliposomes, algal extract, and loaded nanoliposomes are shown in Figure 2. Empty nanoliposomes sample demonstrated its signature peaks of phospholipid at wavenumbers of 3,384 (OH linkage between water and lecithin), 2,927 (CH_2_ asymmetric stretching vibration), 2,858 (CH_2_ symmetric stretching vibration), and 1738 (symmetric stretching vibration of the C = O groups). The peaks related to symmetric and asymmetric stretching of phosphate groups (PO_2_) appeared at wavenumbers of 1,043 and 1,229, respectively (Liu et al., 2018; Liu, Liu, Zhu, Gan, & Le, 2015).

The FTIR spectrum of algal extract showed a strong and wide peak between 3,300 and 3,400/cm (C‐H, O‐H and N‐H stretching), which belongs to main phenolic compounds in the *Sargassum* sp., such as hydroxyl amide and primary amine groups (Kannan, 2014; Moubayed, Al Houri, Al Khulaifi, & Al Farraj, 2017). Other characteristic peaks in the IR spectra of the algal extract appeared at 2,936 (asymmetric stretching of the CH_2_ groups), 1,654 (C = O stretching), 1,463 (C‐O stretching and O‐H bending vibration of carboxylic acids), 1,087 (primary OH groups), 1,023 (S = O stretching vibration), 932, and 883 (C‐H bending), 711 and 629/cm (C‐S stretching) (Kannan, 2014).

When algal extract is loaded in nanoliposomes, some absorption bands changed to higher or lower frequencies, which indicates the interaction of the phenolic compounds with the nanoliposomes bilayers. As shown in Figure 2, the O–H stretching band of loaded nanoliposomes became sharper and shifted to a higher frequency (3,411) compared with algal extract and empty nanoliposomes sample. This change may be due to hydrogen bonds formation between hydroxyl groups of phenolic compounds in the algal extract and the polar head of phospholipids (Tang et al., 2013). The peaks at the frequencies of 2,858/cm and 2,927/cm (symmetric and asymmetric stretching vibration of the CH_2_ groups) in empty nanoliposomes became sharper and displaced to 2,859/cm and 2,929/cm, after loading of phenolic compounds. These alternations confirmed the placement of some phenolic compounds inside bilayer membrane of nanoliposomes (Sarabandi, Sadeghi Mahoonak, et al., 2019b). Moreover, the peak at the wavenumber of 1654 in the algal extract spectrum related to C = O stretching is shifted to a lower frequency (1642) after encapsulation. This indicates the interaction between carbonyl group of phenolic compounds and hydroxyl groups of lecithin through hydrogen bond formation (Rafiee et al., 2017). These results indicated that phenolic compounds are successfully loaded in nanoliposomes and placed near polar head of the phospholipid molecules or even interior regions of bilayers.

### 3.5 DSC studies

The phase transition temperature (Tc) is one of the most effective parameters in the permeability and fluidity of liposome membranes. Generally, due to the impact of this factor on the stability of lipid vesicles, understanding of Tc is important for the manufacture and utilization of liposomes and nanoliposomes (Mozafari, 2010). In present study, the Tc values of empty and loaded nanoliposomes were 1.6 and 6.3°C, respectively.

Increase in phase transition temperature after encapsulation may be caused by the interaction of hydrophobic phenolic compounds and unsaturated phospholipids in lecithin and increase in rigidity of nanoliposomal lipid bilayer. Hydrogen bonds formation between phenolic compounds and polar heads of phospholipids is also effective in improving Tc of liposomes (Cies´lik‐Boczula, Küpcü, Rünzler, Koll, & Köhler, 2009; Tsuchiya, 2010). Other effective parameters on Tc are acyl chain length, degree of saturation of the hydrocarbon chains, particle size, and nature of the encapsulated compound (Rafiee et al., 2017).

### 3.6 Oxidative stability

**Figure 3:**
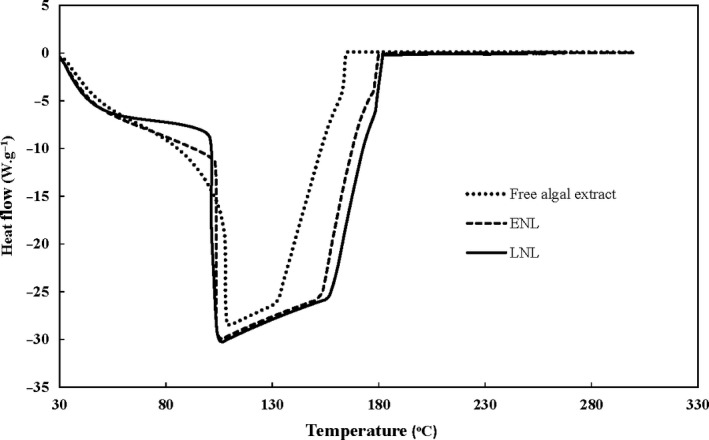
The DSC thermograms of ENL (empty nanoliposomes formulated by 0.5% lecithin at 30°C), free algal extract, and LNL (loaded nanoliposomes manufactured by 0.5% lecithin and 1,313 ppm phenolic compounds at 30°C)

The DSC thermograms can be used to determine thermal‐oxidative stability. By examining the DSC curves, the onset temperature at which the oxidation reaction starts were obtained. As shown in Figure 3, the onset temperatures of free extracts, empty, and loaded nanoliposomes were 132.3, 153.8, and 157.1°C, respectively.

The encapsulated extract showed better oxidative stability than free ones. This result indicates that the extract is incorporated into the nanoliposomes and is protected against decomposition.

Lipid bilayers of liposomes and nanoliposomes are prone to oxidation. The DSC results showed that encapsulating algal extract in nanoliposomes increases oxidative stability of the lipid bilayers. *Sargassum* species have large amounts of phenolic compounds such as meroterpenoids, phlorotannins, and fucoxanthins, and the antioxidant effects of these compounds have reported in many studies (Lim et al., 2019). When algal extract is loaded in nanoliposomes, hydrophilic phenolic compounds scavenge aqueous free radicals near the membrane surface. While hydrophobic polyphenols can penetrate into lipid bilayers, place near unsaturated chains of phospholipids and consequently, reduce the free radicals in lipid bilayers. In addition, the hydrophobic phenolic compounds which located in lipid bilayers increase membrane fluidity and can prevent the propagation of lipid oxidation. Therefore, loading phenolic‐rich extract in nanoliposomes increases the oxidation stability of the nanoliposomes (Fabris, Momo, Ravagnan, & Stevanato, 2008). Similarly, Rafiee et al. (2017) reported that pistachio green hull extract has antioxidant effect on soy lecithin nanoliposomes.

### 3.7 Physical stability

The evaluation of physicochemical properties of nanoliposomes during storage is useful in determining their physical stability. Therefore, the effects of storage of nanoliposomes at 4°C for 2 months on their characteristics were investigated in the present study. As shown in Table 3, particle size did not change significantly up to 15 days. Nevertheless, on subsequent days of storage, the size of the vesicles increased for both empty and loaded nanoliposomes. This small increase in the particle size during storage can be explained considering the possibility of chemical hydrolysis of lipid bilayers and limited aggregation of the vesicles (Nitesh & Ranjan, 2018; Praveen et al., 2019).

**Table 3:** Changes in the characteristics of nanoliposomes during storage at 4°C

Characterestics	Sample	Storage time (d)				
		0	15	30	45	60
Particle size (nm)	ENL	79.1 ± 1.1^h*^	80.5 ± 1.0^h^	83.5 ± 0.8^g^	86.6 ± 2.0^f^	92.9 ± 1.4^e^
	LNL	104.3 ± 0.5^d^	106.0 ± 0.7^d^	109.3 ± 0.9^c^	112.5 ± 1.3^b^	116.1 ± 1.4^a^
PDI	ENL	0.265 ± 0.004^dc^	0.270 ± 0.003^bdac^	0.270 ± 0.002^bdac^	0.275 ± 0.004^ba^	0.277 ± 0.005^a^
	LNL	0.249 ± 0.006^e^	0.262 ± 0.006^d^	0.266 ± 0.005^bdc^	0.267 ± 0.006^bdac^	0.274 ± 0.008^bac^
Zeta potential	ENL	−49.8 ± 0.4^g^	−48.9 ± 0.7^fg^	−47.4 ± 1.3^fe^	−46.7 ± 1.3^e^	−44.4 ± 1.4^d^
	LNL	−41.2 ± 0.6^c^	−39.6 ± 1.5^c^	−35.4 ± 0.7^b^	−34.9 ± 1.2^ba^	−33.4 ± 0.6^a^
Entrapment efficiency	LNL	45.5 ± 1.2^a^	42.1 ± 1.2^b^	37.3 ± 1.1^c^	34.4 ± 1.1^d^	29.5 ± 1.3^e^

* Different letters for each parameter's data represent significant differences among means (LSD test, *p* < .01). ENL (empty nanoliposomes formulated by 0.5% lecithin at 30°C) and LNL (loaded nanoliposomes formulated by 0.5% lecithin and 1,313 ppm of phenolic compounds at 30°C).

The PDI values demonstrated an incremental trend over time. However, these values were below 0.28, indicating the narrow size distribution and physical stability of nanoliposomes during storage.

Zeta potential measurement is useful in determining physical stability of charged particles (Mozafari, 2010). The obtained results showed that with increasing storage time, the zeta potential of nanoliposomes decreases significantly. Compared with empty nanoliposomes, vesicles containing the extract showed a greater reduction in zeta potential. However, after 60 days of storage both empty and loaded nanoliposomes had a zeta potential greater than −30 mV which still indicates an acceptable level of physical stability (Khorasani et al., 2018).

As seen in Table 3, the entrapment efficiency of the phenolic compounds in nanoliposomes decreased over the time and after 60 days reached 29.5%. Generally, because of the thermodynamic instability of liposomes and nanoliposomes, the release of active compounds from these lipid vesicles during storage time is inevitable (Amiri et al., 2018).

### 3.8 In vitro release

**Figure 4:**
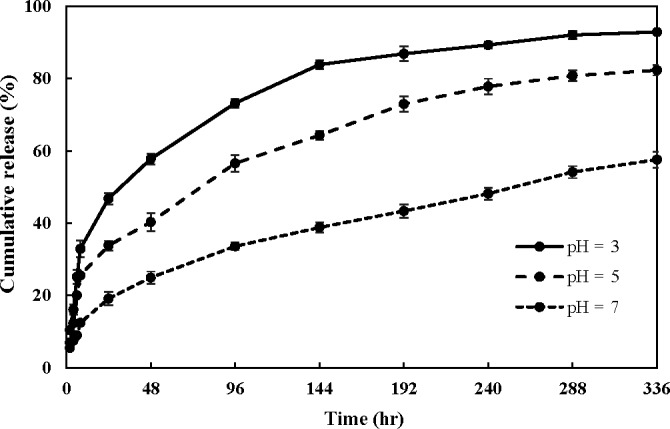
Cumulative release curves for LNL (loaded nanoliposomes formulated by 0.5% lecithin and 1,313 ppm phenolic compounds at 30°C) at different pH values during 336 hr. Error bars represent standard deviation, *n* = 3

The performance of encapsulated bioactive compounds in food systems depends on their release behavior (Rodríguez, Martín, Ruiz, & Clares, 2016). Among nanocarrier systems, nanoliposomes have a high ability to improve targeted and controlled release of bioactive materials (Khorasani et al., 2018). In this study, the in vitro release of phenolic compounds from nanoliposomes was evaluated over time at 25°C and at different pH values. As shown in Figure 4, in all pH values, the release did not occur at a constant rate, and over time, its rate decreased. The initial burst release in the first 8 hr can be related to phenolic compounds entrapped in the external monolayer of the membrane, which can be released more quickly from nanoliposomes (Azzi, Auezova, Danjou, Fourmentin, & Greige‐Gerges, 2018). However, the following slow release may be due to the diffusion of entrapped material from the inner layers to the surface and then diffusion from surface to the bulk of the releasing solution (Lopes, Pinilla, & Brandelli, 2017).

Release of phenolic compounds at pH = 3 was faster than other environments and reached 92.9% after 336 hr, while that was 82.3% and 57.6% at pH = 5 and pH = 7, respectively. At pH = 3, more than 83% of phenolic compounds was released from the nanoliposomes after 144 hr, which was followed by a slow and sustained release. The release pattern at pH = 5 was similar to pH = 3, and its release rate was high up to 10 days and then decreased. Generally, the structure and fluidity of the lipid bilayers are controlled by the pH of the medium, so that acidic pH reduces surface charge of nanoliposomes and decreases the repulsion forces between them, thereby increasing the size of the vesicles. Therefore, the integrity of the phospholipid bilayers is reduced and the release of entrapped material is increased. In the present study, nanoliposomes containing phenolic compounds of the algal extract had high zeta potential and thus were influenced by the pH changes. At pH = 3, the change in the structure of nanoliposomes is greater than pH 5 (Gülseren & Corredig, 2013). Therefore, the burst release of phenolic compounds in acidic pH, especially pH = 3, can be explained.

Since diffusion across lipid bilayers requires that a compound be lipid‐soluble, the ionized form of phenolic compounds cannot get through the membrane. In acidic environments which excess protons are available, the protonated form of phenolic compounds (nonionized) predominates. Therefore, the release of most phenolic compounds, especially at acidic pH, can also be justified from this perspective (Maherani, Arab‐Tehrany, Kheirolomoom, Geny, & Linder, 2013).

Unlike other pH values, any burst release was not observed at pH = 7 and transfer of phenolic compounds from the lipid bilayer was only controlled by diffusion. This sustained and controlled release of phenolic compounds is very important for their use as a food preservative. In agreement with our results, Wang et al. (2017) reported that with decreasing pH, the release of ursolic acid from nanoliposomes increased.

These findings indicate that nanoliposomes are suitable carriers to control the release of phenolic compounds and can maintain the effective levels of these compounds in food systems over a period of time.

### 3.9 Determination of antioxidant activity

In this study, antioxidant activity of free and encapsulated algal extract was also investigated. Table 4 shows the EC_50_ values of free algal extract and algal extract‐loaded nanoliposomes for DPPH^•^, ABTS^•^ **^+^**, and FRAP assays. Ascorbic acid and BHT were considered as positive controls in all tests. According to the data in Table 4, in the DPPH^•^ test, free extract had lower EC_50_ (183.8 ± 5.7) and better antioxidant activity than the encapsulated algal extract. In addition, the results of the ABTS^•^ **^+^** test showed that after the encapsulation in nanoliposomes, the free radical scavenging capacity of the algal extract phenolic compounds decreases.

**Table 4:** Antioxidant activity of free and encapsulated algal extract

Sample	EC_50_ (ppm)		
	DPPH^•^	ABTS^•^ **^+^**	FRAP
Free algal extract	171.4 ± 17.8^b*^	219.5 ± 5.0^b^	129.2 ± 8.8^b^
Encapsulated algal extract	267.7 ± 19.9^c^	453.9 ± 13.8^c^	152.6 ± 6.4^c^
Ascorbic acid	15.8 ± 0.4^a^	96.2 ± 2.4^a^	13.5 ± 0.2^a^
BHT	30.5 ± 1.1^a^	91.4 ± 5.4^a^	33.8 ± 1.4^b^

Abbreviations: ABTS, 2,2'‐azino‐bis (3‐ethylbenzothiazoline‐6‐sulfonic acid); BHT, butylated hydroxytoluene; DPPH, 2,2‐diphenyl‐1‐picrylhydrazyl; FRAP, ferric Reducing Antioxidant Power.

* Different letters within each column represent significant differences among means (LSD test, *p* < .01). EC_50_: the concentration of extract that is required to exert 50% antioxidant activity.

The reducing power is an important mechanism in the antioxidant activity of phenolic compounds (Zou, Liu, et al., 2014a). The effect of encapsulation on the reducing power of algal extract was evaluated by FRAP test and is shown in Table 4. Like the previous two methods, free algal extract showed lower EC_50_ and higher antioxidant activity than loaded nanoliposomes.

Based on the results of antioxidant tests, nanoliposomes had lower antioxidant capacity than free algal extract. The function of bioactive compounds in the lipid vesicles depends on their location and interactions with lipid bilayers. The position of phenolic compounds in the nanoliposomes varies according to their polarity and chemical structure. Some phenolic compounds are absorbed on the surface of the membrane, while others penetrate into lipid bilayers or nanoliposomes core (Mignet, Seguin, & Chabot, 2013). Regarding the diminished antioxidant activity of nanoliposomes compared with the free extract in this study, it can be elucidated that a large proportion of the phenolic compounds of the algal extract are placed inside the core of nanoliposomes. In addition to the hindering effect of the nanoliposomes membrane, weak antioxidant function of the encapsulated phenolic compounds can be explained by their slow release and the interaction of phenolic compounds with the lipid bilayers (Mignet et al., 2013; Zou, Peng, et al., 2014b). This observation is in agreement with Zou, Liu, et al. (2014a), who reported that nanoliposomes containing tea polyphenols have less antioxidant activity than free phenolic compounds. In another study, Zou, Peng, et al. (2014b) found that after encapsulation in nanoliposomes, antioxidant activity of epigallocatechin gallate decreases. These findings are also in agreement with the results of the present study.

## 4 CONCLUSIONS

The algal extract phenolic compounds were successfully encapsulated in soy lecithin nanoliposomes. High zeta potential and narrow particle size of the nanoliposomes indicated a high stability of the formulation during storage. Overall, nanoliposomes encapsulation of algal extract is able to protect the encapsulated material against thermo‐oxidative decomposition. The nanoliposomes have well‐controlled the release of phenolic compounds over time. After encapsulation, a high percentage of antioxidant capacity of the phenolic compounds of the algal extract was maintained. Consequently, encapsulated algal extract can be used as a natural preservative in the production of lipid‐containing foods, especially food emulsions such as margarines and salad dressings.

## CONFLICT OF INTEREST

The authors declare that they have no conflict of interest.

## ETHICAL APPROVAL

On behalf of all coauthors, I, Dr. Mohsen Barzegar, declare that this article has not been published in or is not under consideration for publication elsewhere. All authors were actively involved in the work leading to the manuscript and will hold themselves jointly and individually responsible for its content.
